# Influence of Subclinical Hypothyroidism on Women With Polycystic Ovary Syndrome: A Literature Review

**DOI:** 10.7759/cureus.28468

**Published:** 2022-08-27

**Authors:** Aishwarya Peddemul, Sreedevi Tejovath, Danial Hassan, Khushbu K Patel, Rabia Sikandar, Simranjit S Kahlon, Shaalina Nair, Jihan A Mostafa

**Affiliations:** 1 Obstetrics and Gynecology, California Institute of Behavioral Neurosciences & Psychology, Fairfield, USA; 2 Internal Medicine, California Institute of Behavioral Neurosciences & Psychology, Fairfield, USA; 3 Department of Health Care Profession, Ministry of Public Health, Doha, QAT; 4 Cardiology, California Institute of Behavioral Neurosciences & Psychology, Fairfield, USA

**Keywords:** subclinical hypothyroidism, dyslipidemia, female infertility, homeostatic model assessment of insulin resistance (homa-ir), polycystic ovary syndrome (pcos)

## Abstract

Subclinical hypothyroidism (SCH) is a commonly encountered condition in women with polycystic ovary syndrome (PCOS). Nevertheless, it is unclear whether SCH has any potential impact on the metabolic and reproductive profiles of women with PCOS. Hence, this literature review explores and establishes the link between these two conditions. In women with PCOS, SCH was found to aggravate insulin resistance and dyslipidemia. It was also linked to hormonal imbalances leading to higher infertility rates among the PCOS-SCH group. Therefore, women with PCOS must be screened for thyroid function frequently and managed accordingly.

## Introduction and background

Polycystic ovary syndrome (PCOS) is a commonly encountered endocrine disease affecting 15-20% of females of reproductive age [1]. The reproductive features of PCOS include oligo-anovulation (reduced ovulation), irregular menstrual cycles, and hyperandrogenism (elevated circulating male hormones such as testosterone and dihydrotestosterone) [2]. Hyperandrogenemia is often encountered as hormonal alteration in women with PCOS. It can be due to various reasons, such as disruption in normal ovarian and adrenal function and excess of androgen production. An extra amount of fatty tissue can also contribute to hyperandrogenemia [3]. Decreased aromatase enzyme activity has been partly attributed to the development of hyperandrogenemia in these women. The aromatase enzyme is accountable for the conversion of androgens into estrogens. Inadequate sex hormone-binding globulin (SHBG) levels in women with PCOS can also contribute to elevated levels of circulating androgens [4]. Apart from high androgen levels, most women with PCOS have higher levels of luteinizing hormone and decreased levels of follicle-stimulating hormone, which can result in oligomenorrhea or amenorrhea, multiple tiny cysts on the ovarian surface, and other symptoms such as infertility, hirsutism, and virilization. Hirsutism is seen in 70% of women with PCOS and can be considered a good marker for hyperandrogenism. Other clinical features include alopecia and skin-related problems, including acanthosis nigricans and acne. Since acne is widespread among teenage girls, it should not be considered a marker for PCOS, whereas progressive hirsutism during adolescence can be a consistent marker for PCOS [5,6]. Apart from the effect on the reproductive system, PCOS also affects several metabolic processes in the body, leading to an increased risk of impaired glucose tolerance, prediabetes, type 2 diabetes, metabolic syndrome, and other cardiovascular diseases such as hypertension and hyperlipidemias [7]. Since PCOS has various clinical symptoms, its diagnosis is based on Rotterdam criteria, which require the presence of at least two of the following criteria: (1) oligo/anovulation, (2) hyperandrogenism, and (3) polycystic ovaries on ultrasound. Other etiologies such as congenital adrenal hyperplasia, androgen-secreting tumors, Cushing syndrome, and especially thyroid dysfunction must be excluded before diagnosing PCOS [8].

Hypothyroidism and subclinical hypothyroidism (SCH) (elevated levels of thyroid-stimulating hormone (TSH) with normal free thyroxine) levels are frequently encountered in women with PCOS. It is determined that the rampancy of thyroid disorders in women with PCOS is around 10 %to 15% [9]. There are some similarities in the pathogenesis of both PCOS and Hashimoto's thyroiditis. It can be either due to genetic predisposition or by autoimmune mechanisms mainly attributed to anti-thyroid peroxidase antibodies (TPO) and has been mentioned in a study [10]. Both PCOS and overt hypothyroidism share a similar metabolic profile, including infertility, hyperglycemia (due to insulin resistance), weight gain, and dyslipidemia [11]. Though many women with SCH have mild or no features of thyroid dysfunction, it might lead to or worsen various metabolic and hormonal functions in patients with PCOS [12].

**Figure 1:**
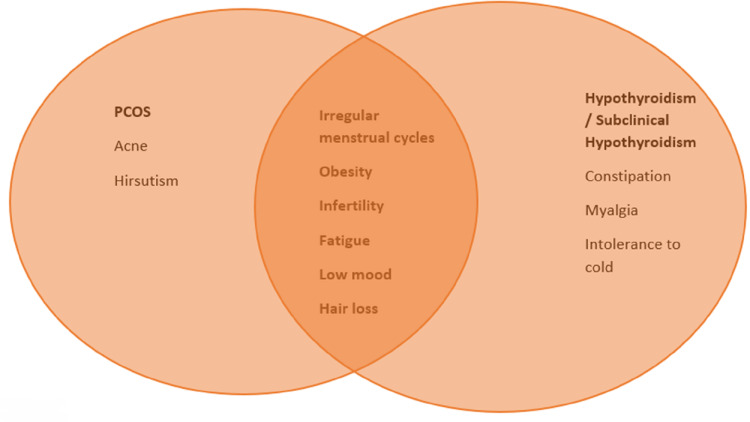
Common clinical findings between PCOS and subclinical hypothyroidism PCOS, polycystic ovary syndrome Figure created by the authors

Individually, PCOS and thyroid disorders exert adverse effects on several metabolic parameters and also the cardiovascular system. However, it remains unclear whether SCH influences the severity of women's metabolic and hormonal profile with PCOS. Hence, this literature review aims to investigate the potential impact of SCH on metabolic parameters of women with PCOS. Some overlapping features between PCOS and SCH have been demonstrated in Figure 1.

## Review

Insulin resistance

**Figure 2:**
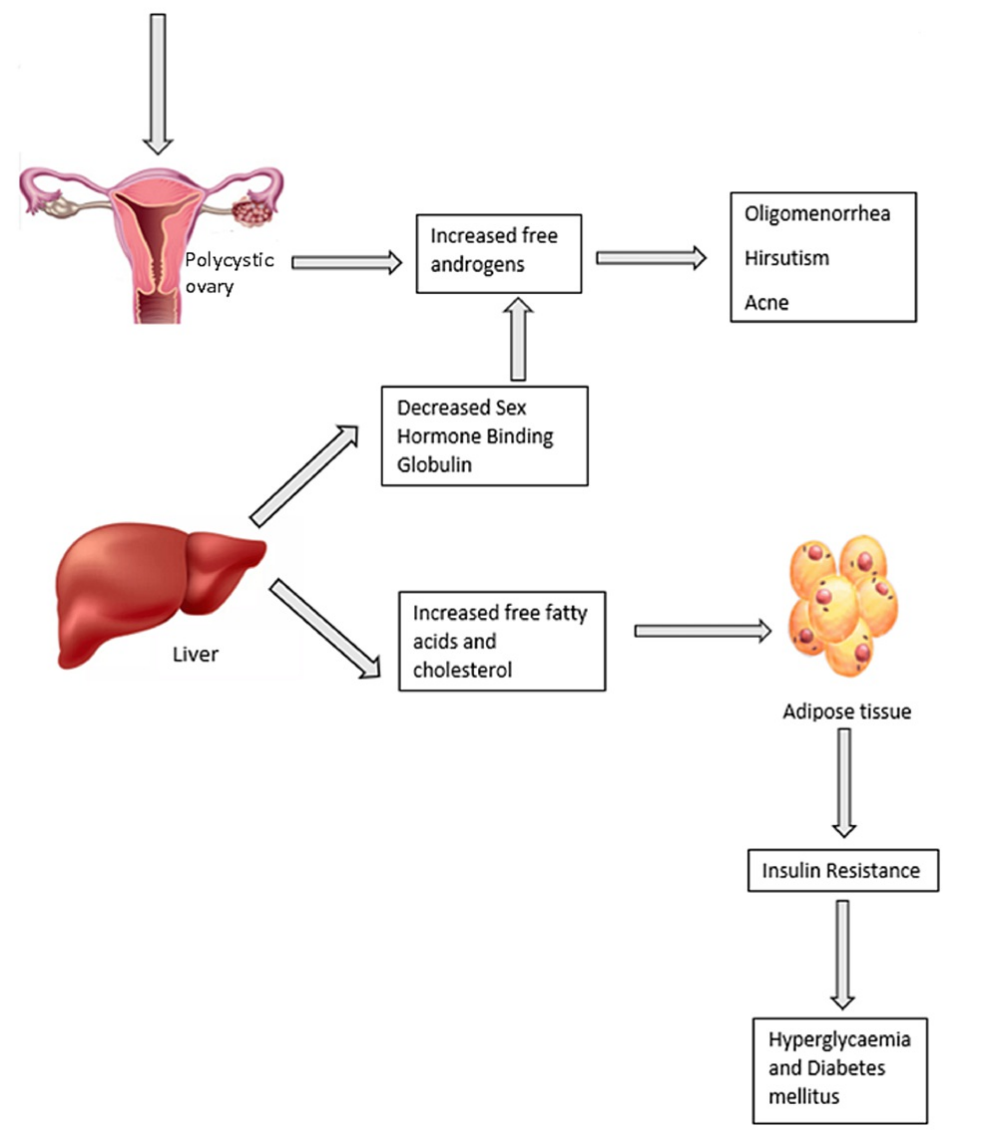
Mechanism of insulin resistance and other clinical features of PCOS PCOS, polycystic ovary syndrome Figure created by the authors

The majority of women with PCOS have insulin resistance, which is independent of their age and body mass index (BMI). It is estimated that the prevalence of insulin resistance in obese women with PCOS is around 70-80% and in lean women with PCOS it is 20-25% [13]. Even though the mechanism for the development of insulin resistance is not clear, it has been attributed to hyperandrogenemia. Other factors such as obesity and thyroid dysfunction may also influence insulin resistance. For example, in the case of hypothyroidism, glucose uptake in muscle and adipose tissue is resistant to insulin, resulting in hyperinsulinemia and insulin resistance [14]. The factors influencing insulin resistance and the mechanism of development of insulin resistance in women with PCOS are as follows. Various genetic and lifestyle factors can also influence PCOS which leads to an increase in free androgens. In women with PCOS, there is an increased production of free fatty acids (FFA) and cholesterol and decreased production of SHBG, leading to insulin resistance, as demonstrated in Figure 2.

Insulin resistance is more common among women with SCH than those without SCH [15], which reveals some association between SCH and insulin resistance; however, the exact mechanism of which is not clear. A significant dependence between TSH value and insulin resistance has been described by Mueller et al. [14] in women with PCOS, irrespective of their BMI [16]. The Homeostatic Model Assessment of Insulin Resistance (HOMA-IR) is an insulin resistance index used to determine a person's chances of developing diabetes; it has been well documented by Mueller et al. in 2009. A study, which included 70 women with TSH values less than 2.5 mlU/l and 33 women with TSH values more than 2.5 mlU/l, revealed that women with PCOS and SCH had an elevated HOMA-IR value and subsequently greater risk of developing diabetes [17]. Another study conducted in 2012 [16], which included 20 women with PCOS and SCH and 39 women with PCOS and euthyroid status, revealed significantly elevated fasting insulin levels and HOMA-IR values. A more recent cross-sectional study (2018), which included 137 females diagnosed with PCOS and having SCH, revealed impaired fasting plasma glucose (FPG) and impaired insulin sensitivity [15]. A meta-analysis conducted in 2018 by de Medeiros et al. [12] revealed significantly elevated FPG values with no significant increase in fasting insulin values and HOMA-IR values. Table 1 demonstrates the association between insulin resistance and SCH in three different studies.

**Table 1:** Association between PCOS-SCH and insulin resistance PCOS, polycystic ovary syndrome; SCH, subclinical hypothyroidism; HOMA-IR, Homeostatic Model Assessment for Insulin Resistance; FPG, fasting plasma glucose [14,15,18]

Author	Publication	Sample size	Finding
Mueller et al. [14]	Oxford Academic	337 women with PCOS	Significant association+
Celik et al. [18]	Taylor & Francis Online	20 women with PCOS and SCH; 39 women with PCOS and normal thyroid function	Significant increase in fasting insulin levels and HOMA-IR values
Bedaiwy et al. [15]	Mary Ann Liebert, Inc.	137 women with PCOS	Among 137 women, 21.9% had SCH and abnormal FPG and HOMA-IR values

From the above, it is clear that SCH in women with PCOS may affect the carbohydrate metabolism leading to significantly higher FPG values, which in the long run may lead to diabetes. However, it is not known whether treating the higher FPG levels would prevent the development of diabetes in these women. A study [19] reported the lowering of TSH value with the usage of metformin and thereby improving insulin sensitivity. Another randomized control trial [20] reported that metformin decreases the TSH levels and decreases insulin resistance if used when TSH levels are more than >2.5 μU/ml. However, further studies are needed to authenticate.

Serum lipids

**Figure 3:**
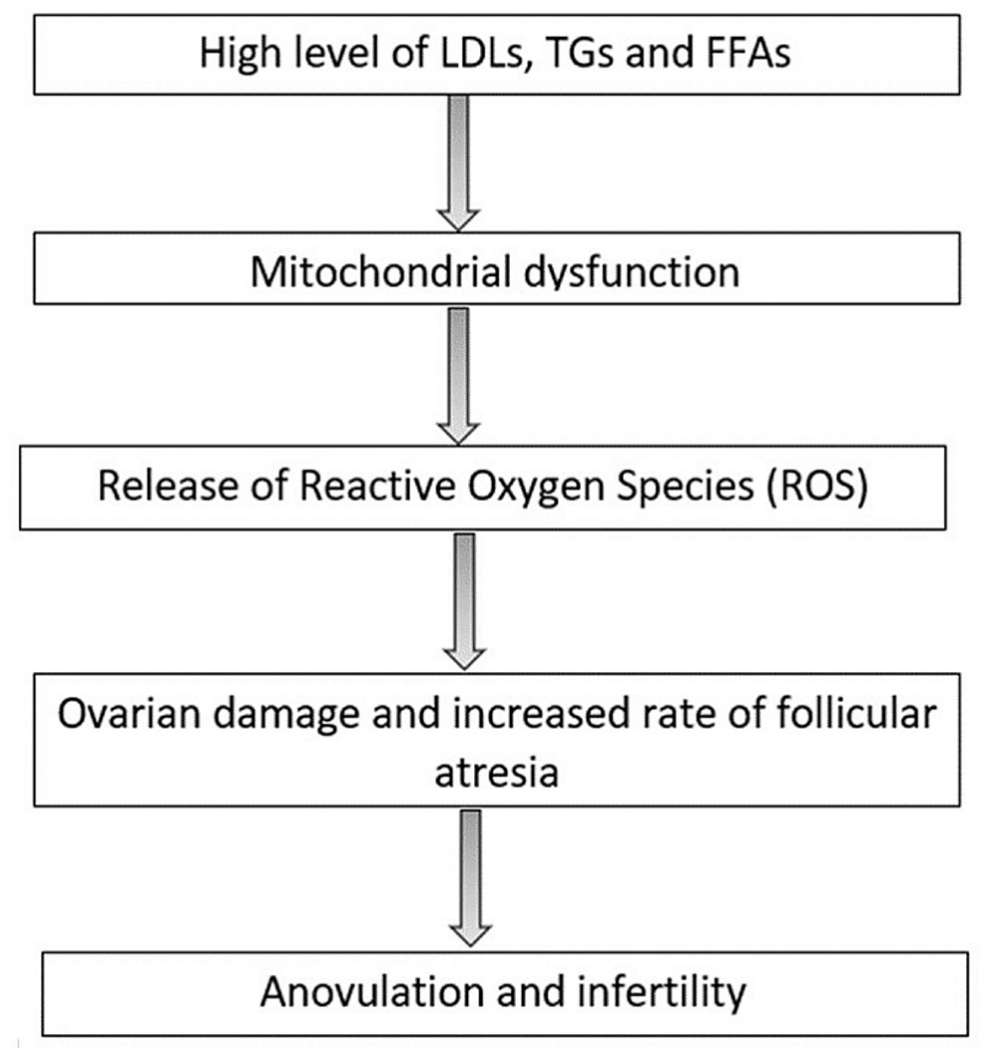
Mechanism of anovulation and infertility in PCOS LDL, low-density lipoprotein; TG, triglycerides; FFA, free fatty acids Figure created by the authors

Dyslipidemia is one of the most commonly encountered metabolic findings in women with PCOS. There is a wide variation in lipid patterns among women with PCOS. This wide variation in lipid patterns may be related to high levels of circulating androgens [21,22]. Mild hypercholesterolemia is a frequently encountered clinical finding in these women and can significantly increase serum triglyceride and lipoprotein levels [23,24]. Dyslipidemia can also cause anovulation by inducing changes in the oocyte environment [25]. High levels of low-density lipoprotein (LDL), triglycerides (TG), and FFA can cause mitochondrial dysfunction by the release of reactive oxygen species, which, in turn, can ovarian damage leading to anovulation and infertility [26-28]. The above-mentioned information has been demonstrated in Figure 3.

Yu and Wang’s study in 2016, which included 100 women with PCOS and 100 control women, revealed altered serum lipid levels and increased frequency of dyslipidemia in women with SCH and PCOS [11]. Apart from SCH, other genetic and environmental factors [29] also play a vital role in developing dyslipidemia. In 2018 [12], a meta-analysis demonstrated higher total cholesterol levels and lower HDL levels in women with SCH and PCOS. Many studies also reported elevated levels of LDL, which is a significant risk factor for coronary artery disease. Though the exact mechanism for the increase in LDL is unclear, it is hypothesized that it might be due to hyperandrogenism or genetic factors [30]. Based on atherogenicity, LDL is subclassified into two types: small dense LDL and large buoyant LDL. Small dense LDL is more atherogenic, increasing the risk of various heart diseases [31]. This small dense LDL is elevated in women with PCOS, increasing the risk of various cardiac problems. TG levels were also significantly higher in women with SCH-PCOS. Similar results were found in a study conducted by Benetti-Pinto et al. in 2013 [32]. The alterations in serum lipid levels including LDL, TG, HDL, and FFA have been demonstrated in Table 2.

**Table 2:** Alterations in serum lipid levels LDL, low-density lipoprotein; HDL, high-density lipoprotein

Type of lipid	Serum levels in women with PCOS and hypothyroidism
LDL	Increased
Triglycerides	Increased
Free fatty acids	Increased
HDL	Decreased

The findings mentioned above show a significant correlation between dyslipidemia and SCH in women with PCOS. Due to the limited studies available and the heterogenicity among the available studies, we should be judicious while treating dyslipidemia in these women. The decision must be taken based on individual circumstances and weighing the benefits and risks. Lifestyle modifications, including a well-balanced diet and exercise, should always be considered a first-line treatment for women with PCOS, especially for those with dyslipidemia.

Infertility

PCOS is one of the most prevalent reasons for anovulatory infertility, and around 90-95% of women seeking anovulatory infertility treatment have PCOS [33]. A study conducted by Benetti-Pinto et al. [32] in 2013 revealed that more than 50% of women with SCH and PCOS had complaints of infertility, and the exact cause could not be explained. However, they could identify higher levels of prolactin hormone in these women, which could be a potential cause of infertility. A higher level of prolactin was explained by the fact that they had elevated thyrotropin-releasing hormone. Similar results were observed in studies conducted by Kaiser [34] and Nizam et al. [35]. A cross-sectional study conducted in 2013 [36] also described worsening infertility in patients with PCOS and hypothyroidism. It was explained that low thyroid hormones interfere with estradiol and progesterone secretion, which, in turn, results in ovarian dysfunction and infertility.

**Figure 4:**
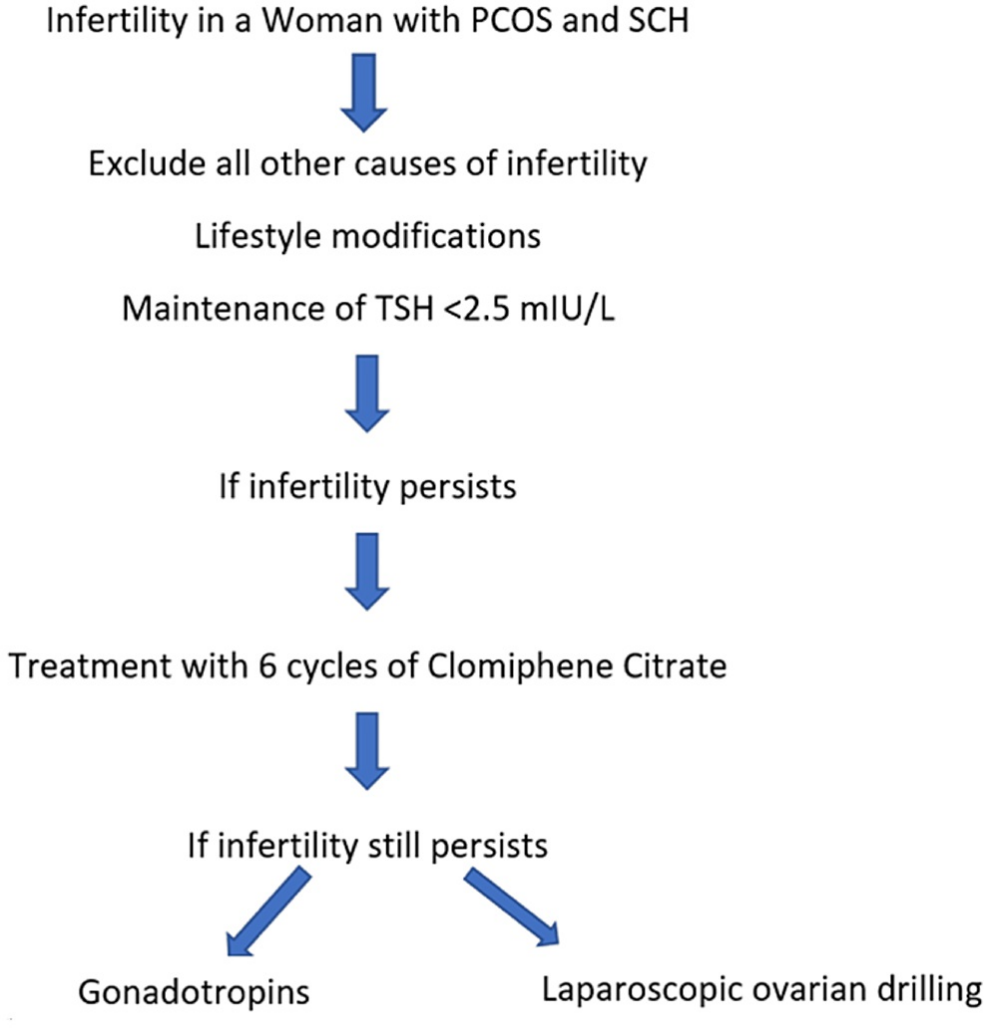
Management of infertility in PCOS PCOS, polycystic ovary syndrome; SCH, subclinical hypothyroidism; TSH, thyroid-stimulating hormone [37-39] Figure created by the authors

Hence, from the findings mentioned above, it is well established that hypothyroidism and SCH can cause hyperprolactinemia leading to infertility, and thus it needs a proper evaluation and management. Evaluation of infertility in women with PCOS should be started six months after attempts to conceive have failed. The principal modality of infertility treatment in women with PCOS and SCH is lifestyle modification. A 5-10% loss in body weight, regardless of BMI, may substantially improve ovulation [37]. In case of failure of lifestyle modifications, pharmacological measures can be implemented. Clomiphene citrate is considered a preferred drug for induction of ovulation. It is usually prescribed to be taken during the early follicular phase [38], and the ovulation rate might reach up to 75% to 80% [39] and subsequently a conception rate of 22% [40]. However, note that the clomiphene citrate treatment must be limited to six ovulatory cycles. Gonadotropins and ovarian drilling can be considered in case of failure of treatment with clomiphene citrate. All other causes including thyroid problems must be addressed before starting the treatment for infertility in women with PCOS. However, this may not be an option in women with autoimmune thyroid disease (who have increased levels of anti-TPO antibodies) because of higher clomiphene citrate resistance [41]. The steps in the management of infertility in a woman with PCOS and SCH have been demonstrated in Figure 4.

Limitations

Though we conducted an extensive literature search in various databases, there could be some limitations in our present study as there are minimal studies on the association between PCOS and SCH. Furthermore, there could be chances of bias due to the heterogeneous nature of the studies. Despite these limitations, we have dramatically improved the credibility of our literature review by pooling the results of various studies available to date.

## Conclusions

Based on the findings of our study, we can conclude that SCH can aggravate insulin resistance, which increases the risk of developing diabetes in the long run in women with PCOS. Metformin can be used to improve insulin sensitivity among these women. Dyslipidemia (increase in total cholesterol levels as well LDLs) was also noted among this group of women, which was statistically significant. Apart from the metabolic alterations, SCH also affects ovulation by causing hyperprolactinemia and thereby causing infertility in these women. Lifestyle modifications can be considered as first-line treatment for all the above-mentioned metabolic and reproductive alterations. Treatment of SCH using metformin can be considered on a case-by-case basis if the benefits outweigh the risks. Due to the limited studies and pieces of evidence, we recommend further studies on this topic, which could benefit many women suffering from PCOS and SCH.

## References

[1] March WA, Moore VM, Willson KJ, Phillips DI, Norman RJ, Davies MJ (2010). The prevalence of polycystic ovary syndrome in a community sample assessed under contrasting diagnostic criteria. Hum Reprod.

[2] Teede HJ, Misso ML, Deeks AA (2011). Assessment and management of polycystic ovary syndrome: summary of an evidence-based guideline. Med J Aust.

[3] Santos BR, Lecke SB, Spritzer PM (2018). Apa-I polymorphism in VDR gene is related to metabolic syndrome in polycystic ovary syndrome: a cross-sectional study. Reprod Biol Endocrinol.

[4] Colditz GA, Peterson LL (2018). Obesity and cancer: evidence, impact, and future directions. Clin Chem.

[5] Fauser BC, Tarlatzis BC, Rebar RW (2012). Consensus on women's health aspects of polycystic ovary syndrome (PCOS): the Amsterdam ESHRE/ASRM-Sponsored 3rd PCOS Consensus Workshop Group. Fertil Steril.

[6] Hsu MI (2013). Changes in the PCOS phenotype with age. Steroids.

[7] Lim SS, Hutchison SK, Van Ryswyk E, Norman RJ, Teede HJ, Moran LJ (2019). Lifestyle changes in women with polycystic ovary syndrome. Cochrane Database Syst Rev.

[8] Kristensen SL, Ramlau-Hansen CH, Ernst E, Olsen SF, Bonde JP, Vested A, Toft G (2010). A very large proportion of young Danish women have polycystic ovaries: is a revision of the Rotterdam criteria needed?. Hum Reprod.

[9] Singla R, Gupta Y, Khemani M, Aggarwal S (2015). Thyroid disorders and polycystic ovary syndrome: an emerging relationship. Indian J Endocrinol Metab.

[10] Arduc A, Aycicek Dogan B, Bilmez S (2015). High prevalence of Hashimoto's thyroiditis in patients with polycystic ovary syndrome: does the imbalance between estradiol and progesterone play a role?. Endocr Res.

[11] Yu Q, Wang JB (2016). Subclinical hypothyroidism in PCOS: impact on presentation, insulin resistance, and cardiovascular risk. Biomed Res Int.

[12] de Medeiros SF, de Medeiros MA, Ormond CM, Barbosa JS, Yamamoto MM (2018). Subclinical hypothyroidism impact on the characteristics of patients with polycystic ovary syndrome. a meta-analysis of observational studies. Gynecol Obstet Invest.

[13] Marshall JC, Dunaif A (2012). Should all women with PCOS be treated for insulin resistance?. Fertil Steril.

[14] Mueller A, Schöfl C, Dittrich R (2009). Thyroid-stimulating hormone is associated with insulin resistance independently of body mass index and age in women with polycystic ovary syndrome. Hum Reprod.

[15] Bedaiwy MA, Abdel-Rahman MY, Tan J (2018). Clinical, hormonal, and metabolic parameters in women with subclinical hypothyroidism and polycystic ovary syndrome: a cross-sectional study. J Womens Health (Larchmt).

[16] Kowalczyk K, Radosz P, Barański K, Pluta D, Kowalczyk D, Franik G, Madej P (2021). The influence of treated and untreated subclinical hypothyroidism on metabolic profile in women with polycystic ovary syndrome. Int J Endocrinol.

[17] Dittrich R, Kajaia N, Cupisti S, Hoffmann I, Beckmann MW, Mueller A (2009). Association of thyroid-stimulating hormone with insulin resistance and androgen parameters in women with PCOS. Reprod Biomed Online.

[18] Celik C, Abali R, Tasdemir N, Guzel S, Yuksel A, Aksu E, Yılmaz M (2012). Is subclinical hypothyroidism contributing dyslipidemia and insulin resistance in women with polycystic ovary syndrome?. Gynecol Endocrinol.

[19] Meng X, Xu S, Chen G, Derwahl M, Liu C (2017). Metformin and thyroid disease. J Endocrinol.

[20] Karimifar M, Aminorroaya A, Amini M, Mirfendereski T, Iraj B, Feizi A, Norozi A (2014). Effect of metformin on thyroid stimulating hormone and thyroid volume in patients with prediabetes: A randomized placebo-controlled clinical trial. J Res Med Sci.

[21] Spałkowska M, Mrozińska S, Gałuszka-Bednarczyk A (2018). The PCOS patients differ in lipid profile according to their phenotypes. Exp Clin Endocrinol Diabetes.

[22] Ramírez-Rodríguez AM, González-Ortiz M, Martínez-Abundis E (2020). Effect of dapagliflozin on insulin secretion and insulin sensitivity in patients with prediabetes. Exp Clin Endocrinol Diabetes.

[23] Kim JJ, Choi YM, Hong MA (2018). Serum visfatin levels in non-obese women with polycystic ovary syndrome and matched controls. Obstet Gynecol Sci.

[24] Tsouma I, Kouskouni E, Demeridou S (2014). Correlation of visfatin levels and lipoprotein lipid profiles in women with polycystic ovary syndrome undergoing ovarian stimulation. Gynecol Endocrinol.

[25] Lin K, Sun X, Wang X, Wang H, Chen X (2020). Circulating adipokine levels in nonobese women with polycystic ovary syndrome and in nonobese control women: a systematic review and meta-analysis. Front Endocrinol (Lausanne).

[26] Schube U, Nowicki M, Jogschies P, Blumenauer V, Bechmann I, Serke H (2014). Resveratrol and desferoxamine protect human OxLDL-treated granulosa cell subtypes from degeneration. J Clin Endocrinol Metab.

[27] Jozkowiak M, Hutchings G, Jankowski M (2020). The stemness of human ovarian granulosa cells and the role of resveratrol in the differentiation of MSCS-a review based on cellular and molecular knowledge. Cells.

[28] Behboudi-Gandevani S, Ramezani Tehrani F, Bidhendi Yarandi R, Noroozzadeh M, Hedayati M, Azizi F (2017). The association between polycystic ovary syndrome, obesity, and the serum concentration of adipokines. J Endocrinol Invest.

[29] Essah PA, Nestler JE, Carmina E (2008). Differences in dyslipidemia between American and Italian women with polycystic ovary syndrome. J Endocrinol Invest.

[30] Kim JJ, Choi YM (2013). Dyslipidemia in women with polycystic ovary syndrome. Obstet Gynecol Sci.

[31] Ivanova EA, Myasoedova VA, Melnichenko AA, Grechko AV, Orekhov AN (2017). Small dense low-density lipoprotein as biomarker for atherosclerotic diseases. Oxid Med Cell Longev.

[32] Benetti-Pinto CL, Berini Piccolo VR, Garmes HM, Teatin Juliato CR (2013). Subclinical hypothyroidism in young women with polycystic ovary syndrome: an analysis of clinical, hormonal, and metabolic parameters. Fertil Steril.

[33] Dennett CC, Simon J (2015). The role of polycystic ovary syndrome in reproductive and metabolic health: overview and approaches for treatment. Diabetes Spectr.

[34] Kaiser UB (2012). Hyperprolactinemia and infertility: new insights. J Clin Invest.

[35] Nizam K, Menon N (2008). Frequency of primary amenorrhea and the outcome of treatment at Liaquat University Hospital. J Liaquat Uni Med Health Sci.

[36] Sinha U, Sinharay K, Saha S, Longkumer TA, Baul SN, Pal SK (2013). Thyroid disorders in polycystic ovarian syndrome subjects: a tertiary hospital based cross-sectional study from Eastern India. Indian J Endocrinol Metab.

[37] Tarlatzis B, Fauser B, Legro RS (2008). Consensus on infertility treatment related to polycystic ovary syndrome. Hum Reprod.

[38] Imani B, Eijkemans M, te Velde E, Habbema J, Fauser B (2002). A nomogram to predict the probability of live birth after clomiphene citrate induction of ovulation in normogonadotropic oligoamenorrheic infertility. Fertil Steril.

[39] Messinis IE (2005). Ovulation induction: a mini review. Hum Reprod.

[40] Eijkemans MJ, Polinder S, Mulders AG, Laven JS, Habbema JD, Fauser BC (2005). Individualized cost-effective conventional ovulation induction treatment in normogonadotrophic anovulatory infertility (WHO group 2). Hum Reprod.

[41] Gaberšček S, Zaletel K, Schwetz V, Pieber T, Obermayer-Pietsch B, Lerchbaum E (2015). Mechanisms in endocrinology: thyroid and polycystic ovary syndrome. Eur J Endocrinol.

